# Psychological Inoculation for Credibility Assessment, Sharing Intention, and Discernment of Misinformation: Systematic Review and Meta-Analysis

**DOI:** 10.2196/49255

**Published:** 2023-08-29

**Authors:** Chang Lu, Bo Hu, Qiang Li, Chao Bi, Xing-Da Ju

**Affiliations:** 1 School of Psychology, Northeast Normal University Changchun City, Jilin Province China; 2 Jilin Provincial Key Laboratory of Cognitive Neuroscience and Brain Development Changchun City, Jilin Province China

**Keywords:** psychological inoculation, misinformation, discernment, sharing, meta-analysis

## Abstract

**Background:**

The prevalence of misinformation poses a substantial threat to individuals’ daily lives, necessitating the deployment of effective remedial approaches. One promising strategy is psychological inoculation, which pre-emptively immunizes individuals against misinformation attacks. However, uncertainties remain regarding the extent to which psychological inoculation effectively enhances the capacity to differentiate between misinformation and real information.

**Objective:**

To reduce the potential risk of misinformation about digital health, this study aims to examine the effectiveness of psychological inoculation in countering misinformation with a focus on several factors, including misinformation credibility assessment, real information credibility assessment, credibility discernment, misinformation sharing intention, real information sharing intention, and sharing discernment.

**Methods:**

Following the Preferred Reporting Items for Systematic Reviews and Meta-Analysis (PRISMA) guidelines, we conducted a meta-analysis by searching 4 databases (Web of Science, APA PsycINFO, Proquest, and PubMed) for empirical studies based on inoculation theory and outcome measure–related misinformation published in the English language. Moderator analyses were used to examine the differences in intervention strategy, intervention type, theme, measurement time, team, and intervention design.

**Results:**

Based on 42 independent studies with 42,530 subjects, we found that psychological inoculation effectively reduces misinformation credibility assessment (*d*=–0.36, 95% CI –0.50 to –0.23; *P*<.001) and improves real information credibility assessment (*d*=0.20, 95% CI 0.06-0.33; *P*=.005) and real information sharing intention (*d*=0.09, 95% CI 0.03-0.16; *P*=.003). However, psychological inoculation does not significantly influence misinformation sharing intention (*d*=–0.35, 95% CI –0.79 to 0.09; *P*=.12). Additionally, we find that psychological inoculation effectively enhances credibility discernment (*d*=0.20, 95% CI 0.13-0.28; *P*<.001) and sharing discernment (*d*=0.18, 95% CI 0.12-0.24; *P*<.001). Regarding health misinformation, psychological inoculation effectively decreases misinformation credibility assessment and misinformation sharing intention. The results of the moderator analyses showed that content-based, passive inoculation was more effective in increasing credibility and sharing intention. The theme of *climate change* demonstrates a stronger effect on real information credibility. Comparing intervention types showed that pre-post interventions are more effective for misinformation credibility assessment, while post-only interventions are better for credibility discernment.

**Conclusions:**

This study indicated that psychological inoculation enhanced individuals’ ability to discern real information from misinformation and share real information. Incorporating psychological inoculation to cultivate an informed public is crucial for societal resilience against misinformation threats in an age of information proliferation. As a scalable and cost-effective intervention strategy, institutions can apply psychological inoculation to mitigate potential misinformation crises.

## Introduction

### Background

Misinformation has become a global problem that threatens people’s daily lives. For example, climate change misinformation confuses the public’s perception of anthropogenic climate change [1], and COVID-19 misinformation prevents people from finding effective treatment strategies [2]. In light of the escalating challenges posed by misinformation, it becomes increasingly evident that honing individuals’ discernment skills is paramount to safeguarding public health and overall well-being.

Misinformation refers to information that is false, but not created with the intention of causing harm [3]. Misinformation is usually measured using self-reports.

In this field of study, researchers present a series of social media posts or news statements containing true or false information to subjects [4,5], after which a binary or Likert scale is used to allow the subjects to assess the information’s credibility and their willingness to share it. However, these methods are somewhat limited. For example, the effect of the intervention may simultaneously reduce the credibility of all the information [6]. Researchers are currently more interested in the concept of “discernment,” which assesses an individual’s ability to discriminate misinformation from real information by calculating the difference between real information and misinformation [7].

### Psychological Inoculation

Studies have provided strategies against misinformation, such as improving social media and filtering algorithms [8,9]. Research has attempted to combat misinformation using a “prebunking” method called psychological inoculation, which confers potential resistance to misinformation before encountering it. Psychological inoculation suggests that the idea of injecting a weakened dose of a virus to activate antibodies can be applied in the context of information processing. It works in 2 ways [10]: motivational threat (the desire to defend against manipulation attacks) and refutational pre-emption (pre-exposure to a weakened example of the attack).

Traditional psychological inoculation research has focused on health behavior promotion, but it is now more focused on misinformation issues [11]. For example, the Bad News game designed by Roozenbeek and van der Linden [5] teaches players about 6 common misinformation strategies by putting them in the role of the creator of misinformation. The result shows that inoculation reduces the perceived reliability and persuasiveness of misinformation. The method has been recognized by the European Commission as the most sustainable means of combating misinformation [12] and has achieved equally significant results in cross-cultural research. Piltch-Loeb et al [13] promoted resistance to COVID-19 vaccine misinformation with a video inoculation. Results found that the inoculated individuals showed greater resistance to misinformation than the uninoculated individuals. Agley et al [14] presented an intervention infographic to prevent COVID-19 misinformation.

### This Study

Recently, there has been some debate about the effectiveness of psychological inoculation in response to misinformation. Some researchers argue that psychological inoculation reduces the belief in all information and that the ability to effectively discriminate between real and false information is not improved [15]. Additionally, the field has moved from “content-based” to “technology-based” immunization, and the application of active (vs passive) inoculations [10]. However, the effectiveness of these strategies requires further investigation [16].

Based on these concerns, this study conducted a meta-analysis to investigate the effectiveness of psychological inoculation in combating misinformation, focusing on the influence of misinformation credibility assessment, real information credibility assessment, credibility discernment, misinformation sharing intention, real information sharing intention, and sharing discernment. In addition, we further investigated the effectiveness of psychological inoculation in response to health misinformation. During COVID-19, the increasing risk of misinformation, such as vaccination hesitancy driven by misinformation [17,18], created a new challenge for general public health in the online setting. Comprehending the efficacy of psychological inoculation as a response to misinformation can establish a reliable foundation for subsequent policy implementation.

### Potential Moderators

Many study characteristics influence the intervention effect [19]. We examined the following moderators: intervention strategy, intervention type, theme, measurement time, team, and intervention design.

Intervention strategies and types may have an impact on the effects of psychological inoculation. For the intervention strategies, content-based inoculation focuses on the content of the information, and technology-based inoculation focuses on the technology behind the information [10]. Whether technology-based inoculation can achieve a similar effect to content-based inoculation needs to be further tested. For the intervention type, passive inoculation requires that participants be asked to read the rebuttals provided in the inoculation information without actively engaging in generating their own responses [20]. Active inoculation requires participants to generate their own rebuttals in response to the rebuttal information provided. The cognitive processes involved in active inoculation, which involves “internal” rebuttals, are more effective than passive inoculation [21]. In this study, graphics and courses were classified as passive inoculation, while videos and games were classified as active inoculation because they were more likely to prompt people to generate responses.

Misinformation interventions commonly engage with different themes, with current major themes including climate change, policy, and health [1,14,22]; whether these interventions are effective for additional issues also needs to be investigated.

The durability of misinformation is an important indicator of intervention effectiveness and when additional intervention is needed [23,24]. Therefore, the difference in measurement time of psychological inoculation needs to be clarified. Based on the existing study, we divided the measurement time into “immediately,” “1-week follow-up,” and “2 weeks or more” [25].

The team of authors may also influence the effectiveness of psychological inoculation. Van der Linden’s team [1] introduced psychological inoculation into the misinformation issue and has conducted many related studies with his collaborators [24,26-28]. We used van der Linden’s team and other researchers as a moderator to examine the reliability of the psychological inoculation effect.

Despite the existence of a control group, different intervention designs may still affect intervention results. For example, the intervention effects may be different in the post-only test condition than in the pre-post test condition [27]. We distinguished between these 2 intervention designs to validate the inoculation effect.

## Methods

### Literature Retrieval

**Figure 1:**
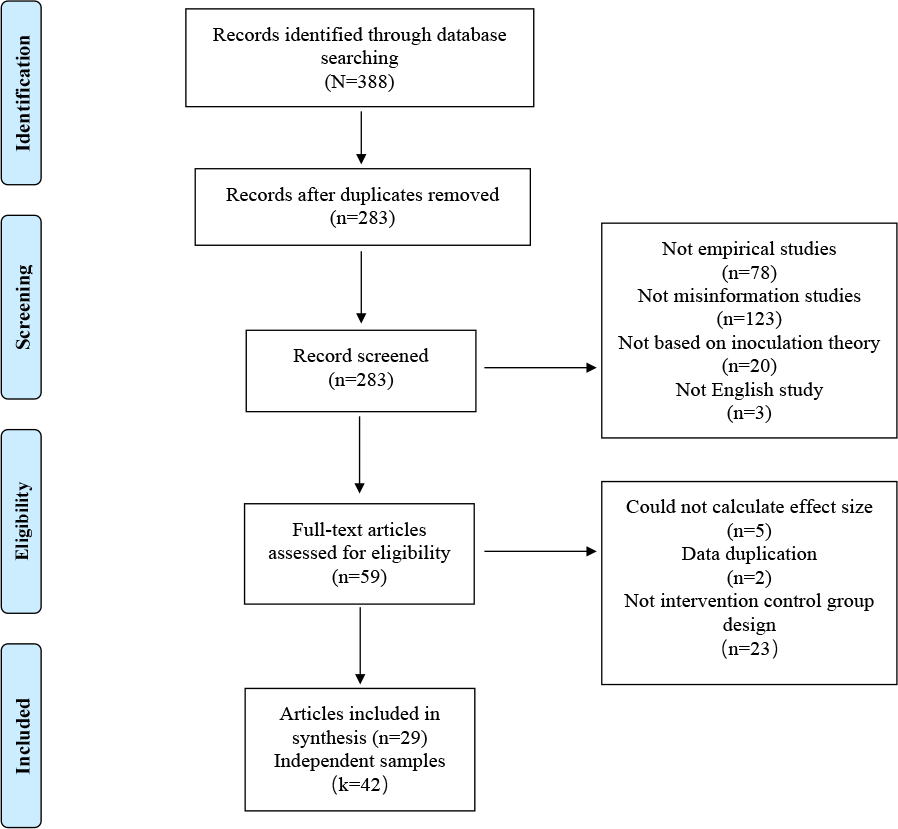
PRISMA (Preferred Reporting Items for Systematic Reviews and Meta-Analyses) flowchart of the study selection process; *n* refers to the number of articles, while *k* refers to the number of studies independently sampled in these articles.

This meta-analysis followed the PRISMA (Preferred Reporting Items for Systematic Reviews and Meta-Analysis) guidelines (see Multimedia Appendix 1) for identifying studies [29]. A literature search was conducted in electronic databases including Web of Science, APA PsycINFO, Proquest, and PubMed. The following 2 search strings were combined using Boolean search terms in titles, abstracts, and keywords: (“Inoculating” OR “Inoculation”) AND (“misinformation” OR “disinformation” OR “conspiracy theor*” OR “fake news” OR “rumor” OR “false information”). Specific search strategies for different databases can be accessed in Multimedia Appendix 2. The initial database search was conducted until April 2023. A summary of the study flow is shown in Figure 1.

### Inclusion Criteria

The inclusion and exclusion criteria of this study are shown in Textbox 1.

**Inclusion and exclusion criteria.**

**Inclusion criteria**

- Studies covering people who may have been exposed to misinformation
- Studies based on inoculation theory that included a control group for comparison
- Studies that included a placebo, no inoculation intervention, or no intervention as a control
- Studies with the outcomes of misinformation credibility assessment, real information credibility assessment, credibility discernment, misinformation sharing intention, real information sharing intention, and sharing discernment
- Studies measuring assessment of information, changes in attitudes, or behavioral intentions
- Studies that included randomized controlled trials
- Empirical, peer-reviewed studies
- Studies providing effect size or data to enable the calculation of effect sizes
- Studies written in English

**Exclusion criteria**

- Studies including children younger than 6 years; these require adult supervision according to European Union regulations
- Studies not based on inoculation theory
- Studies not including a control group
- Studies not related to credibility assessment and sharing intention for real information or misinformation
- Studies including non–randomized controlled trials
- Reviews and nonempirical studies
- Studies not providing enough data to enable the calculation of effect sizes
- Studies not written in English

### Data Extraction

Relevant data were extracted from the selected studies, including study information (first author and year of publication), sample size, study country, sample age, misinformation theme, intervention type (active vs passive), intervention strategy (content-based vs technology-based), measurement, measurement time, team (van der Linden’s team vs others), intervention design (post only and pre-post), and outcome (misinformation credibility assessment, real information credibility assessment, credibility discernment, misinformation sharing intention, real information sharing intention, and sharing discernment).

### Quality Assessment

The Cochrane Risk of Bias 2 tool (RoB 2.0) was used to assess intervention studies [30]. Funnel plots, the Egger regression test [31], the precision-effect test (PET), and the precision-effect estimate with SE (PET-PEESE) were used to examine publication bias in intervention studies [32], and the Duval and Tweedie [33] trim-and-fill method was used to adjust for publication bias. A leave-one-out sensitivity analysis was performed to ensure that no single study significantly influenced the results. Two independent coders assessed the quality of the studies according to the criteria of these tools. There were no significant differences between the 2 coders.

### Data Analysis

In this study, the effect size is calculated using the Cohen *d*, which refers to a standardized difference in means standardized by the change in SD. The Cohen *d* was calculated using R (R Core Team). For each study, means, SDs, and sample size were extracted. If the study did not report statistics, the Cohen *d* was calculated using *χ*^2^, *t*, η, and *F* values. The Cohen *d* distinguishment criteria were as follows: 0.2 for a small effect size, 0.5 for a medium effect size, and 0.8 for a large effect size [34]. A negative value indicated lower credibility assessment and sharing intention for misinformation and real information compared to the control conditions. A positive value indicated a higher discernment ability for credibility and sharing compared to the control conditions. The *Q* statistic and *I*^2^ statistic were used to estimate heterogeneity and a random effect model was used to combine effect sizes. Moderator analyses were used to examine potential moderators that might influence intervention effects [35]. All analyses were conducted using the *meta* (version 6.2-1), *dmeta* (version 0.0.9000), and *metafor* (version 3.8-1) R packages [36-38]. A *P* value <.05 was considered statistically significant.

## Results

A total of 29 articles that included 42 independent studies with a total sample size of 42,530 were included in this meta-analysis. Specific information on all included studies can be accessed in Multimedia Appendix 3. Research on psychological inoculation against misinformation was mainly published from 2017 to 2023, with most studies conducted with immediate measurement (k=35). Major misinformation themes included health (k=15) and climate change (k=7), the mean participant age distribution was 18 to 48 years, and most studies were conducted in the United States (k=28). A total of 31 studies reported misinformation credibility assessment [7,13,14,16,22-24,26-28,39-48], 26 reported real information credibility assessment [1,7,14-16,22-24,27,41-43,49-54], 12 reported credibility discernment [7,23,24,27,41], 12 reported misinformation sharing intention [7,13,23,40,45,50], 11 reported real information sharing intention [7,23,51,55], and 8 studies reported sharing discernment [7,23].

### Misinformation Credibility Assessment

**Figure 2:**
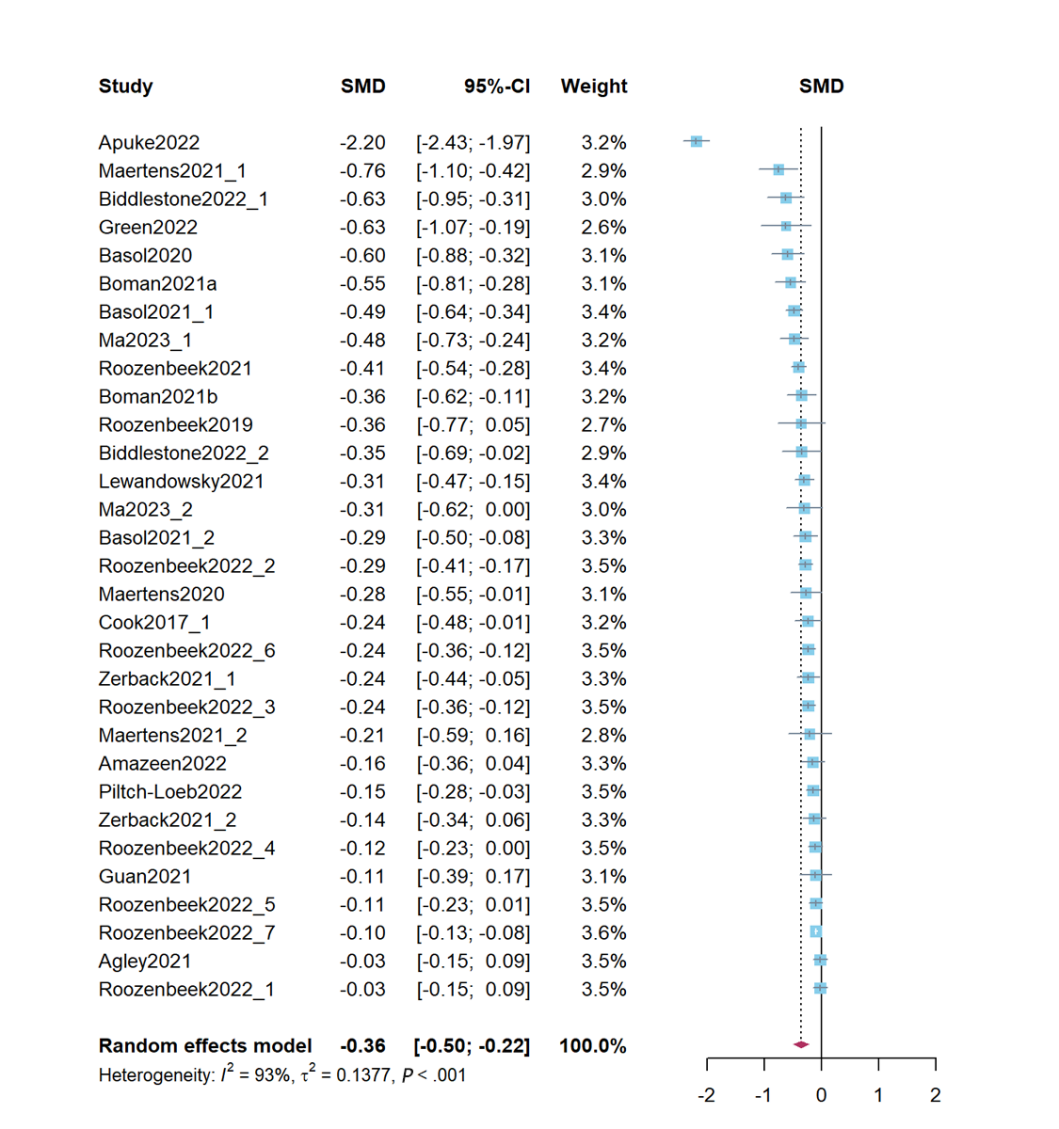
Forest plot for the effects of psychological inoculation on misinformation credibility (k=31). Articles published by the same author in the same year are distinguished by *a* and *b*. Different studies in the same article are distinguished by an underscore and the numbers 1 to 7 [7,13,14,16,22-24,26-28,39-48]. SMD: standardized mean difference.

Combined studies on reducing misinformation credibility assessment revealed that psychological inoculation effectively reduced misinformation credibility (*d*=–0.36, 95% CI –0.50 to –0.23; *P*<.001). The heterogeneity test results were significant (*Q*=433.32; *P*<.001; *I*^2^=93.1%; τ^2^=0.14; τ=0.37). Figure 2 presents a forest plot of the effect sizes. Sensitivity analysis revealed effect sizes ranging from *d*=–0.37 (95% CI –0.51 to –0.23; *P*<.001) to *d*=–0.27 (95% CI –0.34 to –0.21; *P*<.001), demonstrating stability of the results.

### Real Information Credibility Assessment

**Figure 3:**
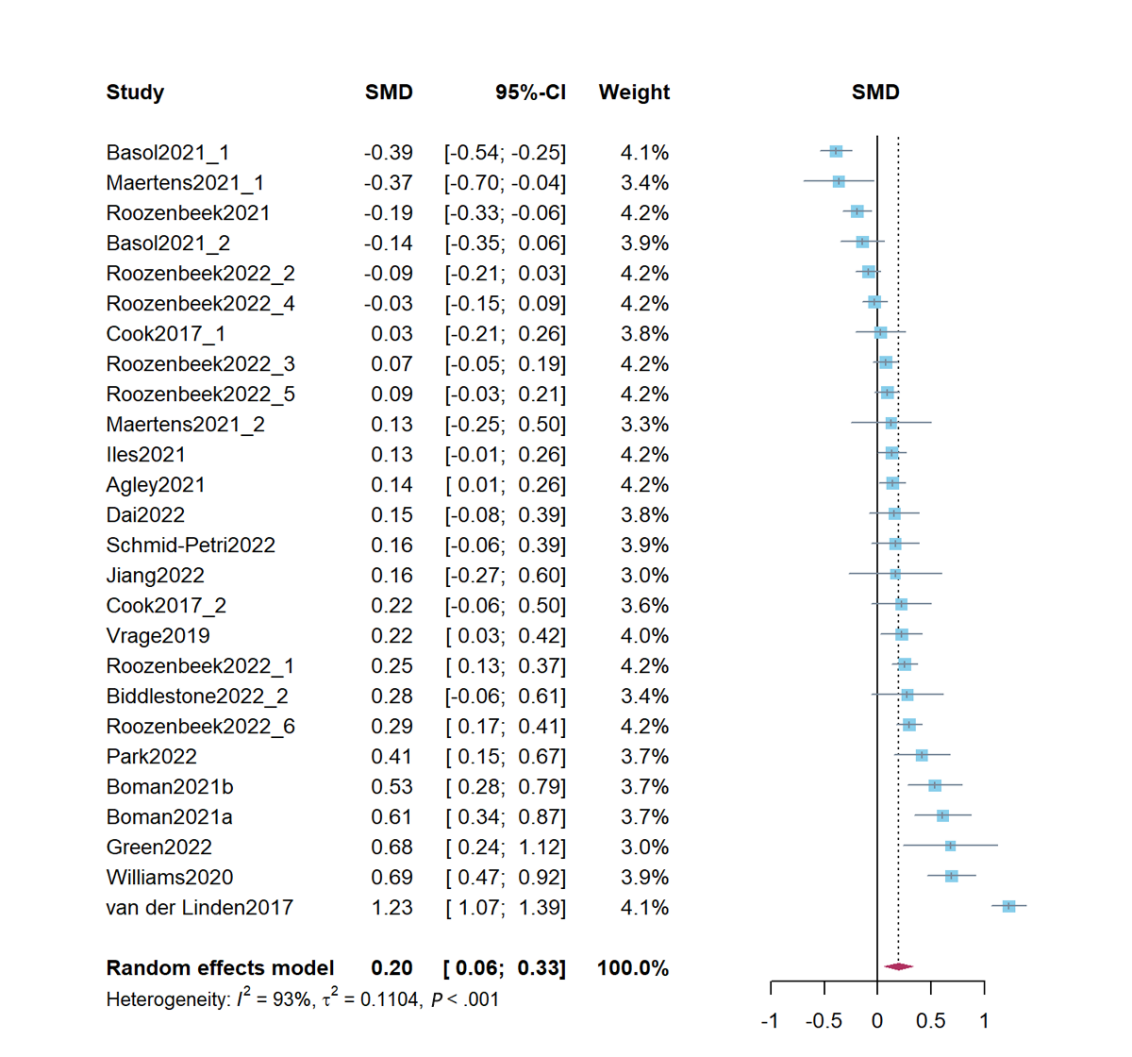
Forest plot for the effects of psychological inoculation on real information credibility (k=26). Articles published by the same author in the same year are distinguished by *a* and *b*. Different studies in the same article are distinguished by an underscore and the numbers 1 to 6 [1,7,14-16,22-24,27,41-43,49-54]. SMD: standardized mean difference.

Combined studies on improving real information credibility revealed that psychological inoculation effectively improved real information credibility (*d*=0.20, 95% CI 0.06-0.33; *P*=.005). The heterogeneity test results were significant (*Q*=355.08; *P*<.001; *I*^2^=93%; τ^2^=0.11; τ=0.33). Figure 3 presents a forest plot of the effect sizes. Sensitivity analysis revealed effect sizes ranging from *d*=0.15 (95% CI 0.04-0.26; *P*=.007) to *d*=0.22 (95% CI 0.09-0.35; *P*=.001), demonstrating stability of the results.

### Credibility Discernment

**Figure 4:**
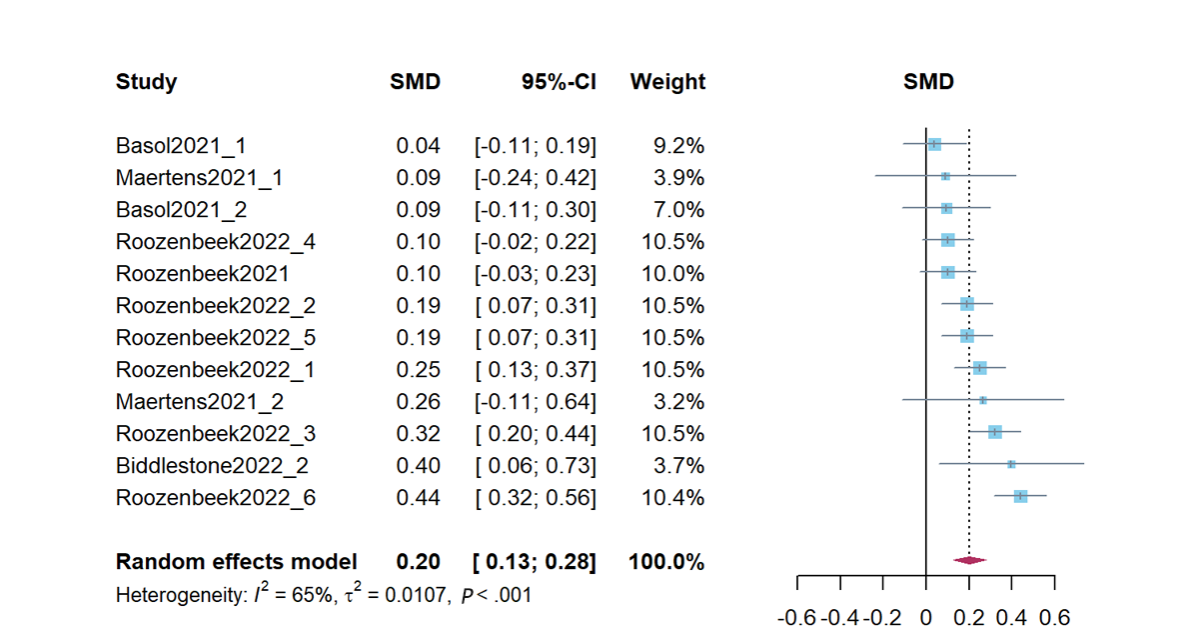
Forest plot for the effects of psychological inoculation on credibility discernment (k=12). Different studies in the same article are distinguished by an underscore and the numbers 1 to 6 [7,23,24,27,41]. SMD: standardized mean difference.

Combined studies on improving credibility discernment revealed that psychological inoculation effectively improved credibility discernment (*d*=0.20, 95% CI 0.13-0.28; *P*<.001). The heterogeneity test results were significant (*Q*=31.74; *P*<.001; *I*^2^=65.3%; τ^2^=0.01; τ=0.10). Figure 4 presents a forest plot of the effect sizes. Sensitivity analysis revealed effect sizes ranging from *d*=0.18 (95% CI 0.11-0.24; *P*<.001) to *d*=0.22 (95% CI 0.14-0.30; *P*<.001), demonstrating stability of the results.

### Misinformation Sharing Intention

**Figure 5:**
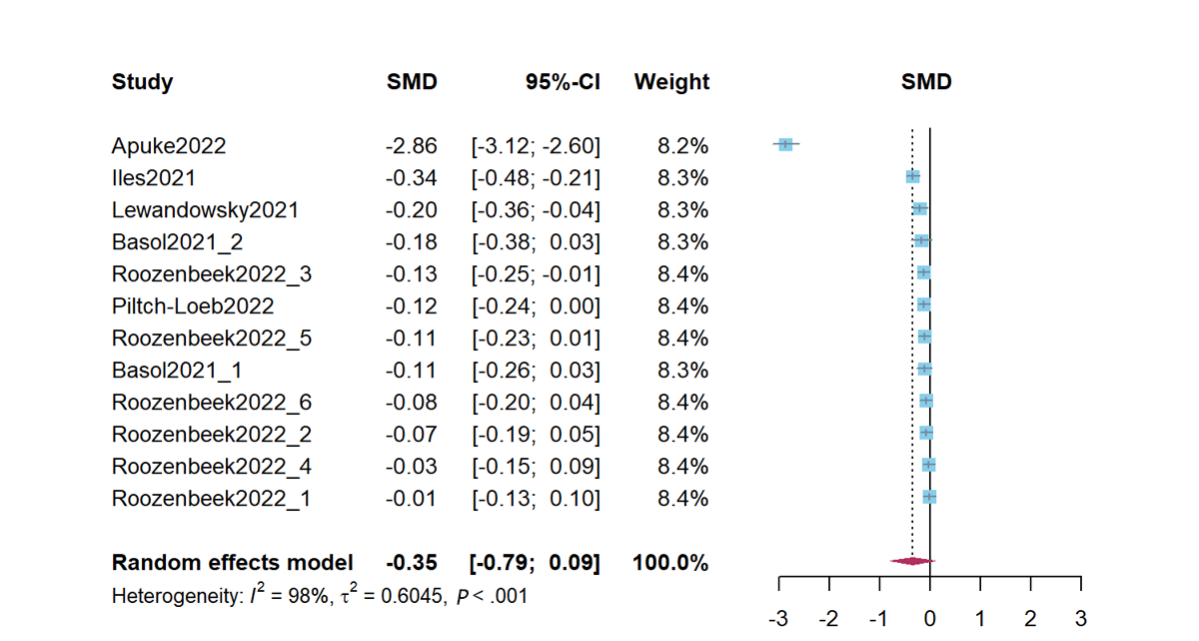
Forest plot for the effects of psychological inoculation on misinformation sharing intention (k=12). Different studies in the same article are distinguished by an underscore and the numbers 1 to 6 [7,13,23,40,45,50]. SMD: standardized mean difference.

Combined studies on reducing misinformation sharing intention revealed that psychological inoculation did not effectively reduce misinformation sharing intention (*d*=–0.35, 95% CI –0.79 to 0.09; *P*=.12). The heterogeneity test results were significant (*Q*=448.88; *P*<.001; *I*^2^=97.5%; τ^2^=0.60; τ=0.77). Figure 5 presents a forest plot of the effect sizes. Sensitivity analysis revealed effect sizes ranging from *d*=–0.38 (95% CI –0.86 to 0.10; *P*=.11) to *d*=–0.12 (95% CI –0.17 to –0.07; *P*<.001).

### Real Information Sharing Intention

**Figure 6:**
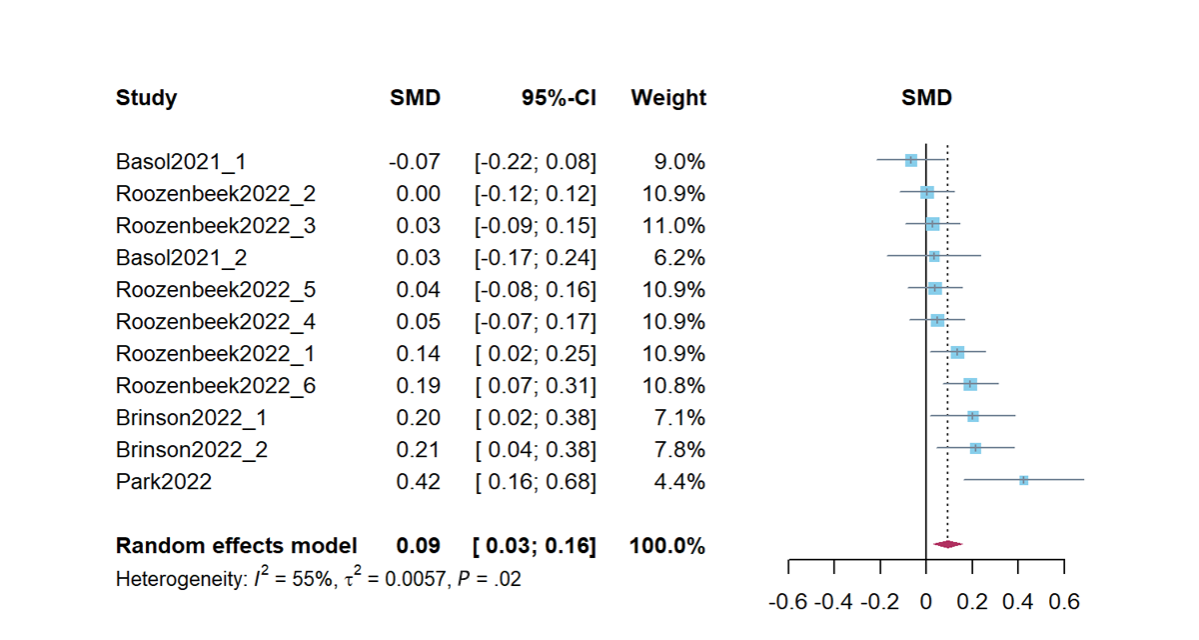
Forest plot for the effects of psychological inoculation on real information sharing intention (k=11). Different studies in the same article are distinguished by an underscore and the numbers 1 to 6 [7,23,51,55]. SMD: standardized mean difference.

Combined studies on improving real information sharing intention revealed that psychological inoculation effectively improved real information sharing intention (*d*=0.09, 95% CI 0.03-0.16; *P*=.003). The heterogeneity test results were significant (*Q*=22.02; *P*=.02; *I*^2^=54.6%; τ^2^=0.01; τ=0.08). Figure 6 presents a forest plot of the effect sizes. Sensitivity analysis revealed effect sizes ranging from *d*=0.08 (95% CI 0.12-0.13; *P*=.007) to *d*=0.11 (95% CI 0.05-0.17; *P*<.001).

### Sharing Discernment

**Figure 7:**
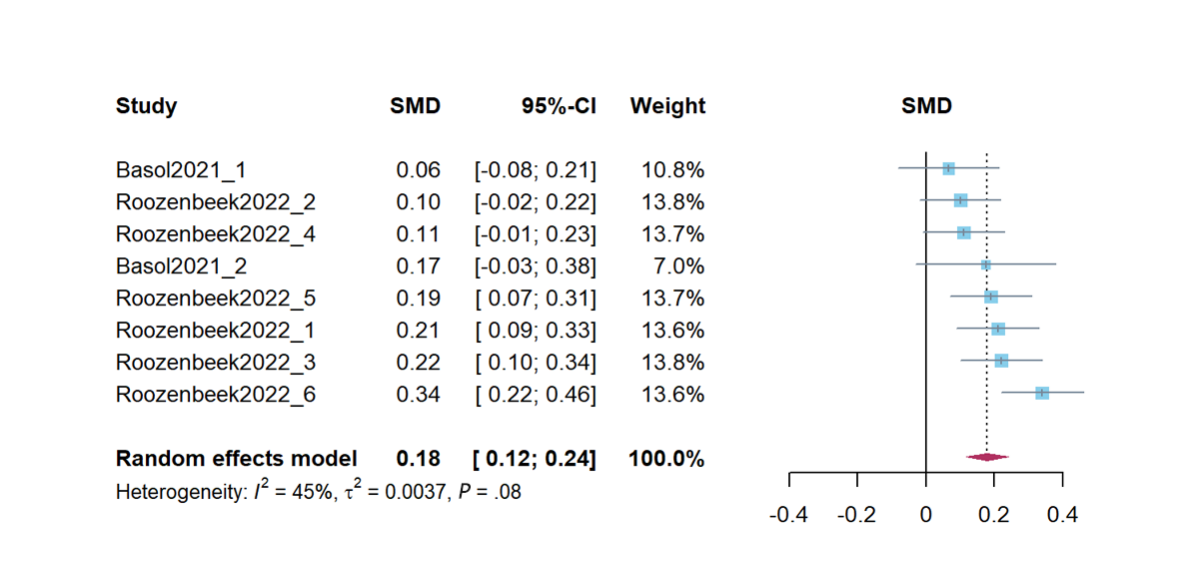
Forest plot for the effects of psychological inoculation on sharing discernment (k=8). Different studies in the same article are distinguished by an underscore and the numbers 1 to 6 [7,23]. SMD: standardized mean difference.

Combined studies on improving sharing discernment revealed that psychological inoculation effectively improved sharing discernment (*d*=0.18, 95% CI 0.12-0.24; *P*<.001). The results of the heterogeneity test were marginally significant (*Q*=12.82; *P*=.08, *I*^2^=45.4%, τ^2^=0.01, τ=0.06). Figure 7 presents a forest plot of the effect sizes. Sensitivity analysis revealed effect sizes ranging from *d*=0.15 (95% CI 0.11-0.20; *P*<.001) to *d*=0.19 (95% CI 0.13-0.26; *P*<.001).

### Psychological Inoculation Against Health Misinformation

Due to the public concern about health misinformation after COVID-19, we further analyzed the effectiveness of psychological inoculation in combating health misinformation. We included studies that focused only on the health theme of *misinformation* (studies with multiple themes were not included). A total of 15 independent studies with 6732 subjects were included. Studies were published from 2021 to 2023, implying that psychological inoculation responded to health misinformation that emerged with the outbreak of COVID-19. The mean age of the participants was 36 years. The main study countries were the United States (k=10) and China (k=3), and the studies were conducted mainly with graphics (k=13).

In combined studies of health misinformation, we found that psychological inoculation significantly reduced the health misinformation credibility assessment (*d*=0.28, 95% CI –0.41 to –0.15; *P*<.001; k=9) and sharing intention (*d*=–0.19, 95% CI –0.30 to –0.08; *P*=.001; k=4). However, psychological inoculation did not have an effect on health real information credibility assessment (*d*=0.05, 95% CI–0.15 to 0.25; *P*=.61; k=7), sharing intention (*d*=0.15, 95% CI –0.01 to 0.30; *P*=.07; k=5), credibility discernment (*d*=0.06, 95% CI –0.06 to 0.18; *P*=.34; k=2), or sharing discernment (*d*=0.10, 95% CI –0.02 to 0.22; *P*=.09; k=2).

### Moderator Analyses

Moderator analyses were used to examine differences in intervention strategies, intervention types, themes, measurement time, teams, and intervention designs (Table 1). Subgroups of moderators included in fewer than 3 studies were excluded from the analysis [56].

The intervention type had no significant effect on misinformation credibility assessment (*Q*=1.23; *P*=.26) or sharing intention (*Q*=1.33; *P*=.25), but had a significant effect on real information credibility assessment (*Q*=13.22; *P*<.001) and sharing intention (*Q*=8.98; *P*=.003). The intervention effect was better with passive inoculation than active inoculation.

Intervention strategy had no significant effect on misinformation credibility assessment (*Q*=1.70; *P*=.19) but had a significant effect on real information credibility assessment (*Q*=7.81; *P*=.005) and sharing intention (*Q*=8.98; *P*=.003). Intervention effects were better for the content-based inoculations than the technology-based inoculations.

The intervention theme had no significant effect on misinformation credibility assessment (*Q*=0.84; *P*=.10), misinformation sharing intention (*Q*=0.49; *P*=.78), or real information sharing intention (*Q*=0.72; *P*=.40), but it had a significant effect on real information credibility assessment (*Q*=25.52; *P*<.001). The *climate change* theme was more effective than the *politics* and *health* themes.

Intervention design had no significant effect on real information credibility assessment (*Q*=0.08; *P*=.78) or misinformation sharing intention (*Q*=1.06; *P*=.30) but had a significant effect on misinformation credibility assessment (*Q*=3.84; *P*=.05) and credibility discernment (*Q*=7.19; *P*=.007). Pre-post interventions were more effective than post-only interventions for misinformation credibility assessment. However, post-only interventions were better than pre-post interventions for credibility discernment.

Measurement time had no significant effect on misinformation credibility assessment (*Q*=4.13; *P*=.13). Study team had no significant effect on misinformation credibility assessment (*Q*=0.66; *P*=.42), real information credibility assessment (*Q*=2.63; *P*=.11), or misinformation sharing intention (*Q*=1.45; *P*=.23).

**Table 1:** Moderator analysis of psychological inoculation against misinformation for different intervention strategies, intervention types, themes, measurement time, teams, and intervention designs.

Outcome and moderator				Cohen *d* (95% CI)		k		*Q*		*P* value		*I*^2^, %
**Misinformation credibility assessment**												
	Main effect		–0.36 *(*–0.50 to –0.22)		31		N/A^a^		N/A		93	
	**Intervention type**							1.26		.26		
		Active	–0.27 (–0.36 to –0.18)		15						84	
		Passive	–0.43 (–0.68 to –0.17)		16						95	
	**Intervention strategy**							1.70		.19		
		Content	–0.26 (–0.36 to –0.15)		10						60	
		Technology	–0.41 (–0.60 to –0.21)		21						95	
	**Theme**							0.84		.10		
		Health	–0.28 (–0.41 to –0.15)		9						77	
		Policy	–0.30 (–0.41 to –0.18)		5						38	
		Climate	–0.31 (–0.48 to –0.18)		3						15	
		Other^b^	–0.43 (–0.71 to –0.14)		14						97	
	**Measuring time**							4.13		.12		
		Immediately	–0.37 (–0.54 to –0.19)		24						94	
		One week	–0.43 (–0.62 to –0.23)		4						55	
		Two weeks or more	–0.19 (–0.34 to –0.04)		3						0	
	**Team**							0.66		.42		
		Van der Linden	–0.29 (–0.39 to –0.20)		16						84	
		Other	–0.41 (–0.68 to –0.14)		15						95	
	**Intervention design**							3.84		.05		
		Post only	–0.21 (–0.28 to –0.15)		19						69	
		Pre-post	–0.53 (–0.85 to –0.22)		12						96	
**Real information credibility assessment**												
	Main effect		0.20 (0.06 to 0.33)		26		N/A		N/A		93	
	**Intervention type**							13.22		<.001		
		Active	–0.03 (–0.16 to 0.11)		11						89	
		Passive	0.38 (0.20 to 0.55)		15						92	
	**Intervention strategy**							7.81		.005		
		Content	0.40 (0.18 to 0.62)		11						93	
		Technology	0.04 (–0.08 to 0.17)		15						87	
	**Theme**							25.52		.001		
		Health	0.05 (–0.15 to 0.25)		7						88	
		Policy	0.06 (–0.05 to 0.18)		2						0	
		Climate	0.57 (0.39 to 0.75)		6						95	
		Other^b^	0.50 (0.13 to 0.88)		11						83	
	**Team**							2.63		.11		
		Van der Linden	0.09 (–0.14 to 0.31)		13						96	
		Other	0.30 (0.18 to 0.42)		13						72	
	**Intervention design**							0.08		.78		
		Post only	0.20 (0.11 to 0.29)		18						75	
		Pre-post	0.14 (–0.26 to 0.54)		8						98	
**Credibility discernment**												
	Main effect		0.20 (0.13 to 0.28)		12		N/A		N/A		65	
	**Intervention design**							7.19		.007		
		Post only	0.26 (0.17 to 0.35)		7						69	
		Pre-post	0.09 (0.00 to 0.17)		5						0	
**Misinformation sharing intention**												
	Main effect		–0.35 (–0.79 to 0.09)		12		N/A		N/A		98	
	**Intervention type**							1.33		.25		
		Active	–0.09 (–0.14 to –0.05)		9						0	
		Passive	–1.11 (–2.83 to 0.62)		3						99	
	**Theme**							0.49		.78		
		Health	–0.19 (–0.30 to –0.08)		4						58	
		Policy	–0.20 (–0.36 to –0.04)		1							
		Other^b^	–0.47 (–1.24 to 0.31)		7						99	
	**Team**							1.45		.23		
		Van der Linden	–0.08 (–0.13 to –0.04)		8						0	
		Other	–0.88 (–2.17 to 0.41)		4						99	
	**Intervention design**							1.06		.30		
		Post only	–0.12 (–0.18 to 0.06)		9						54	
		Pre-post	–1.05 (–2.82 to 0.72)		3						99	
**Real information sharing intention**												
	Main effect		0.09 (0.09 to 0.16)		11		N/A		N/A		55	
	**Intervention type**							8.98		.003		
		Active	0.06 (–0.00 to 0.11)		3						33	
		Passive	0.25 (0.14 to 0.36)		8						7	
	**Intervention strategy**							8.98		.003		
		Content	0.25 (0.14 to 0.36)		8						7	
		Technology	0.06 (–0.00 to 0.11)		3						33	
	**Theme**							0.72		.40		
		Health	0.15 (–0.01 to 0.30)		5						71	
		Other	0.07 (0.01 to 0.13)		6						30	

^a^N/A: not applicable.

^b^Other included hybrid themes or studies without a theme.

### Publication Bias

**Figure 8:**
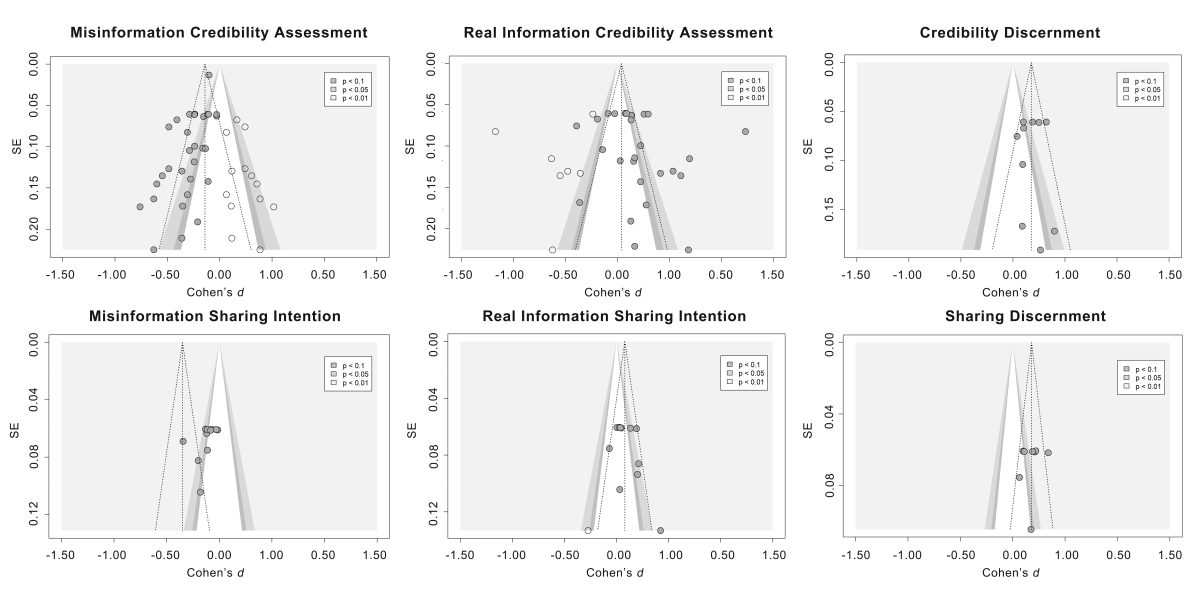
Funnel plot of the relationship between effect sizes and their SEs by different outcomes. Solid dots indicate the effect size and SE of the included studies, and hollow dots represent studies that were added by the trim-and-fill method.

Publication bias was examined using funnel plots (Figure 8), the Egger test, and the PET-PEESE test.

In studies on psychological inoculation for misinformation credibility assessment, the Egger test (1-tailed *t* values) was significant (t_29_=–3.27; *P*=.003), indicating a significant publication bias. The effect size was adjusted to *d*=–0.14 (95% CI –0.30 to –0.01; *P*=.07) after adding 14 studies using the Duval and Tweedie trim-and-fill method; the results were not significant. The intercept in the PET-PEESE test was not significant; we interpret this as showing that the estimate corrected for publication bias (β=–.14; t_29_=–1.32; *P*=.20).

In studies on psychological inoculation for real information credibility assessment, the Egger test was not significant (t_24_=1.13; *P*=.27), indicating no significant publication bias. The effect size was adjusted to *d*=0.04 (95% CI –0.12 to 0.19; *P*=.07) after adding 7 studies using the Duval and Tweedie trim-and-fill method; the results were marginally significant. The intercept in the PET was not significant; we interpret this as showing that the estimate corrected for publication bias (β=–.01; t_24_=–0.06; *P*=.95).

In studies on psychological inoculation for credibility discernment, the Egger test was not significant (t_10_=–0.26; *P*=.80), indicating no significant publication bias. There were no additional studies needed using the Duval and Tweedie trim-and-fill method. The intercept in the PEESE test was significant and can be considered as showing that the effect size estimate corrected for publication bias (β=0.21; t_10_=4.03; *P*=.002).

In studies on psychological inoculation for misinformation sharing intention, the Egger test was significant (t_10_=–3.69; *P*=.004), indicating a significant publication bias. There were no additional studies needed using the Duval and Tweedie trim-and-fill method. The intercept in the PET was not significant and can be considered as showing that the effect size estimate corrected for publication bias (β=1.04; t_10_=1.36; *P*=.20).

In studies on psychological inoculation for real information sharing intention, the Egger test was not significant (*t*_9_=1.63; *P*=.14), indicating no significant publication bias. The effect size was adjusted to *d*=0.08 (95% CI 0.01-0.15; *P*=.03) after adding 1 study using the Duval and Tweedie trim-and-fill method; the results remained the same. The intercept in the PET was not significant; we interpret this as showing that the estimate corrected for publication bias (β=–.12; *t*_9_=–0.92; *P*=.38).

In studies on psychological inoculation for sharing discernment, the Egger test was not significant (*t*_6_=–0.46; *P*=.66), indicating no significant publication bias. There were no additional studies needed using the Duval and Tweedie trim-and-fill method. The intercept in the PEESE test was significant and can be considered as showing that the effect size estimate corrected for publication bias (β=0.28; *t*_6_=1.33; *P*=.23).

## Discussion

### Principal Findings

In this study, we conducted a meta-analysis to examine the effectiveness of psychological inoculation against misinformation. Based on 42 independent studies, we found that psychological inoculation was effective in reducing misinformation credibility assessment and improving real information credibility assessment as well as real information sharing intention. However, psychological inoculation did not effectively influence misinformation sharing intention. For discernment, we found that psychological inoculation was effective in improving ability for credibility discernment and sharing discernment.

### The Effectiveness of Psychological Inoculation Against Misinformation

First, psychological inoculation was effective in reducing misinformation credibility assessment (*d*=–0.36) and improving real information credibility assessment (*d*=0.20) and real information sharing intention (*d*=0.09). This is consistent with existing research that psychological inoculation increases people’s immunity to misinformation and makes them less likely to believe the misinformation they subsequently encounter [5,57]. Second, psychological inoculation did not effectively influence misinformation sharing intention (*P*=.12). This indicates that the effectiveness of psychological inoculation in reducing the dissemination of misinformation is moderate. A possible reason is that individuals sharing misinformation are influenced by other factors [58,59]. Last, psychological inoculation was effective in improving abilities for credibility discernment (*d*=0.20) and sharing discernment (*d*=0.18). Contrary to the opinion that psychological inoculation reduces the credibility of all information [6], psychological inoculation improves individuals’ ability to distinguish between real information and misinformation [7]. This extends psychological inoculation’s practical implications. These results suggest that psychological inoculation is an effective method for helping the public cope with the misinformation they receive.

### The Effectiveness of Psychological Inoculation Against Health Misinformation

Psychological inoculation also effectively decreased misinformation credibility assessment (*d*=0.28) and sharing intention (*d*=–0.19). This is consistent with previous studies [13], indicating the efficacy of psychological inoculation in helping the public cope with the risk of health misinformation. However, psychological inoculation did not influence real information credibility assessment and sharing intention or credibility discernment and sharing discernment. This demonstrates that psychological inoculation was insufficient to boost public trust in real information on health.

### Moderator Analysis

First, different intervention types and intervention strategies did not affect credibility and sharing intention for misinformation, but content-based and passive inoculation were more effective in increasing credibility and sharing intention for real information. A possible reason is that interventions place more focus on reliable statements themselves when performing the inoculation, which leads to better results. Second, the theme of *climate change* had a better effect on real information credibility after inoculation, which implies the great potential of psychological inoculation to respond to the climate change crisis. Third, there was no significant difference in the measurement time, which implies that psychological inoculation may have good durability [24]. Fourth, van der Linden’s team did not differ significantly from other researchers, implying that different research teams arrived at similar results, increasing the reliability and generalizability of the results. Finally, the intervention design influenced the intervention effect for misinformation credibility assessment and credibility discernment. Pre-post interventions were more effective than post-only interventions for misinformation credibility assessment. However, the post-only interventions were better than pre-post interventions for credibility discernment. Intervention effects may be magnified because the post-only condition does not assess preintervention status. Similarly, in the pre-post condition studies, there may have been a practice effect among subjects who answered the same questions and thus achieved better results [27].

### Limitations

Current research on countering misinformation through psychological inoculation has some limitations, and future research needs to expand its scope. First, most of the current studies did not examine decay trends and only assessed the durability of intervention effects at separate time points. Future studies could assess effects at more time points to clarify when the best effects are reached after intervention and when they decay rapidly. Second, most of the studies were conducted in Western countries through online interventions. There is a lack of testing of the effects in a standardized laboratory setting, and cultural representativeness has also not been considered. Future studies should examine the effects of psychological inoculation in standard laboratory conditions and test them in Eastern countries. Finally, most current studies have been conducted with adults and representative populations, although older adults and adolescents tend to be more susceptible to misinformation [60,61]. Future studies need to focus on these susceptible groups.

### Conclusion

In conclusion, this study demonstrates that psychological inoculation is an effective approach against misinformation. It effectively strengthens individuals’ discernment abilities, enabling them to distinguish between genuine and false information. Furthermore, when applied to health misinformation, psychological inoculation shows promise in diminishing credibility assessment and sharing intention for health-related falsehoods, contributing to safeguarding public health. These findings have profound implications for public health and digital well-being. Psychological inoculation can be easily scaled to a broad population at a low cost, and institutions can apply it to mitigate potential misinformation crises [7]. Incorporating psychological inoculation strategies into public health initiatives and educational programs can empower individuals to make well-informed decisions in an era characterized by information proliferation. Cultivating a discerning and well-informed public is crucial to building societal resilience against the pervasive threats posed by misinformation.

## Appendix

Multimedia Appendix 1 PRISMA (Preferred Reporting Items for Systematic Reviews and Meta-Analysis) checklist.

Multimedia Appendix 2 Search strategies.

Multimedia Appendix 3 Characteristics of studies included in the meta-analysis.

## Abbreviations

PET-PEESE: precision-effect estimate with standard error
PET: precision-effect test
PRISMA: Preferred Reporting Items for Systematic Reviews and Meta-Analysis
RoB 2.0: Cochrane Risk of Bias 2 tool
SMD: standardized mean difference

## Data Availability

The data sets generated during and/or analyzed during this study are available in the Open Science Framework repository [62].
