# Supplementary material for: Psychological Inoculation for Credibility Assessment, Sharing Intention, and Discernment of Misinformation: Systematic Review and Meta-Analysis
Source: J Med Internet Res. 2023 Aug 29;25:e49255. doi: 10.2196/49255 (PMC10498317; doi:10.2196/49255)
Supplement: Multimedia Appendix 2 [file jmir_v25i1e49255_app2.docx]

## Multimedia Appendix 1: Search Strategies

Web of Science

Inoculating or Inoculation (Topic) and misinformation or disinformation or conspiracy theor or fake news or rumor or false information (Topic)

APA PsycInfo

S1 = AB ( misinformation or disinformation or conspiracy theor or fake news or rumor or false information ) AND AB ( Inoculating or Inoculation )

S2 = TI ( misinformation or disinformation or conspiracy theor or fake news or rumor or false information ) AND TI ( Inoculating or Inoculation )

S3 =(S1 OR S2)

Proquest

(ti(misinformation OR disinformation OR conspiracy theor OR fake news OR rumor OR false information) AND ti(Inoculating OR Inoculation)) OR (su(misinformation OR disinformation OR conspiracy theor OR fake news OR rumor OR false information) AND su(Inoculating OR Inoculation))

PubMed

(Inoculating[Title/Abstract]ORInoculation[Title/Abstract])AND(misinformation[Title/Abstract] OR disinformation[Title/Abstract] OR conspiracy theor[Title/Abstract] OR fake news[Title/Abstract] OR rumor[Title/Abstract] OR false information[Title/Abstract])
